# Assessing Usability of Smartwatch Digital Health Devices for Home Blood Pressure Monitoring among Glaucoma Patients

**DOI:** 10.3390/informatics9040079

**Published:** 2022-10-06

**Authors:** Sonali B. Bhanvadia, Manreet S. Brar, Arash Delavar, Kiana Tavakoli, Bharanidharan Radha Saseendrakumar, Robert N. Weinreb, Linda M. Zangwill, Sally L. Baxter

**Affiliations:** 1 Hamilton Glaucoma Center, Division of Ophthalmology Informatics and Data Science, Viterbi Family Department of Ophthalmology, Shiley Eye Institute, University of California San Diego, La Jolla, CA 92093, USA; 2 Health Department of Biomedical Informatics, University of California San Diego, La Jolla, CA 92093, USA

**Keywords:** glaucoma, blood pressure, monitoring, usability, user interface, digital health, patient-generated data, gerontechnology, inclusive design

## Abstract

Glaucoma is a leading cause of blindness worldwide. Blood pressure (BP) dysregulation is a known risk factor, and home-based BP monitoring is increasingly used, but the usability of digital health devices to measure BP among glaucoma patients is not well studied. There may be particular usability challenges among this group, given that glaucoma disproportionately affects the elderly and can cause visual impairment. Therefore, the goal of this mixed-methods study was to assess the usability of a smart watch digital health device for home BP monitoring among glaucoma patients. Adult participants were recruited and given a smartwatch blood pressure monitor for at-home use. The eHEALS questionnaire was used to determine baseline digital health literacy. After a week of use, participants assessed the usability of the BP monitor and related mobile app using the Post-study System Usability Questionnaire (PSSUQ) and the System Usability Scale (SUS), standardized instruments to measure usability in health information technology interventions. Variations in scores were evaluated using ANOVA and open-ended responses about participants’ experience were analyzed thematically. Overall, usability scores corresponded to the 80th–84th percentile, although older patients endorsed significantly worse usability based on quantitative scores and additionally provided qualitative feedback describing some difficulty using the device. Usability for older patients should be considered in the design of digital health devices for glaucoma given their disproportionate burden of disease and challenges in navigating digital health technologies, although the overall high usability scores for the device demonstrates promise for future clinical applications in glaucoma risk stratification.

## 1. Introduction

Glaucoma is a progressive optic neuropathy and is the leading cause of irreversible blindness worldwide [1]. Nearly 80 million individuals are estimated to be affected, which is only expected to rise with an aging global population [2,3]. The pathophysiology of glaucoma is complex and not completely understood [1]. Intraocular pressure (IOP) plays a role for many patients and is currently the only proven modifiable risk factor [3]. However, up to 50% of patients with high IOP never develop glaucoma [1], suggesting other risk factors play a role [4].

Vascular conditions such as hypertension, diabetes, and coronary artery disease have been hypothesized to have a role in glaucoma development and progression [5]. Several population-based cross-sectional studies, such as the Rotterdam Eye Study [6] and the Egna-Neumarkt Glaucoma Study [4], have demonstrated an association between elevated blood pressure, elevated IOP, and glaucoma. The Blue Mountains Eye Study [7] also demonstrated that systemic hypertension is related to an increased risk of glaucoma and found that this elevated risk was independent of the effect of elevated BP on raising IOP. However, the relationship between BP and glaucoma is multifaceted, as the Barbados Eye Studies showed that lower systolic BP was also associated with risk of developing glaucoma [8]. Several subsequent studies found that hypotension is a risk factor for glaucoma, and specifically a reduction in BP at night, known as nocturnal dipping, appears to make the optic nerve more susceptible to damage [9].

Because BP fluctuates throughout the day and night and is very situationally dependent, elucidating its complex relationship with a chronic disease such as glaucoma is challenging [10]. Traditional sources of clinical data, such as electronic health records (EHRs), rarely include nighttime BPs for ambulatory patients. The current standard for phenotyping circadian BP regulation is cuff-based ambulatory BP monitoring, which is not used in routine clinical practice due to its cost and difficulty of use [11–14]. However, in recent years, digital health devices have been developed to make home-based circadian BP monitoring more feasible, and data generated by these devices can be useful for patients, clinicians, and researchers seeking to understand the relationship between circadian regulation of BP and risk of glaucoma. One such tool is the Omron Heartguide (Omron, Kyoto, Japan), the first smartwatch for BP monitoring approved by the United States Food and Drug Administration [15], which became commercially available in 2019. While these digital health devices offer great potential, real-world use can be challenging, particularly for elderly and visually impaired individuals. In this study, we assessed the usability of this device in order to better understand these challenges and inform future efforts to make these devices more inclusive to glaucoma patients—a group that is older and has less digital literacy than the population as a whole [16]. Our primary objective was to evaluate overall usability, and secondary aims included understanding whether usability varied by demographic characteristics such as age, gender, or race. To our knowledge, no prior studies have specifically evaluated the usability of these BP smartwatch monitors in individuals with glaucoma.

## 2. Materials and Methods

### 2.1. Study Population

This study was approved by the Institutional Review Board at the University of California San Diego (UCSD) and adhered to the tenets of the Declaration of Helsinki. Participants were recruited at UCSD from the Diagnostic Innovations in Glaucoma Study (DIGS) [17], a general investigation examining various diagnostic technologies in glaucoma, and The African Descent and Glaucoma Evaluation Study (ADAGES) [18], which focuses on patients of African descent, a traditionally under-represented group in clinical research studies. All DIGS/ADAGES participants were required to be 18 years old or older, and for this ancillary study we preferentially selected participants over the age of 40, given our specific interest in digital health use among older individuals. Specific inclusion and exclusion criteria for DIGS/ADAGES have been described in detail previously [17,18]. In brief, participants were required to have at least one eye with open angles and best corrected visual acuity of 20/40 or better to be included. Participants taking a medication known to affect visual field sensitivity and eyes with a history of intraocular surgery (except uncomplicated glaucoma and cataract surgery), a secondary cause of elevated intraocular pressure, a coexisting intraocular disease affecting visual field, or a problem other than glaucoma affecting color vision were excluded.

Additional exclusion criteria for this study included wrist circumference less than 5.3 in (13.5 cm) or greater than 8.5 in (25 cm) (based on manufacturer guidelines for the BP monitor), or cognitive or physical impairment precluding the use of a wristwatch device or a mobile app. Per the manufacturer guidelines, individuals were also excluded from enrollment if they had a wrist injury on the side that the watch would be worn on, need for vascular access or therapy (such as AV shunts), current need for intravenous drip or blood transfusion, proximity to high-frequency devices, need for oxygen-enriched environments, or known diagnosis of heart rhythm problems, arteriosclerosis, or conditions causing poor perfusion of blood flow, problems with motion or trembling, anticipated air travel during the study period, or current pregnancy of history of pre-eclampsia. There were no eligibility criteria based on gender, race, or socioeconomic status.

### 2.2. Study Procedures and Data Collection

At the enrollment visit, participants were asked to complete the standardized eHealth Literacy Scale (eHEALS), which is a validated 8-item questionnaire that measures a patient’s baseline digital health literacy [19], characterized by knowledge, comfort, and perceived skills at finding, evaluating, and applying electronic health information to health problems. They were also asked baseline questions in regard to their glaucoma diagnosis and demographics.

During this visit, each participant was trained by a research assistant on how to use the Omron BP monitor smartwatch, including how to fit it properly to their wrist, how to take BP readings, and how to pair the watch to the Omron mobile application on their phone via Bluetooth. The Omron BP watch is a commercially available medical device approved by the US Food & Drug Administration (FDA) that gives clinically accurate blood pressure readings. It functions as a typical smartwatch, but also tracks heart rate, blood pressure, steps, and sleep cycles. Like a typical BP cuff, it has an inflatable cuff and takes oscillometric measurements. The readings using the device have been found to be within 2 mmHg of measurements using a standard mercury sphygmomanometer for both systolic and diastolic blood pressure [15]. A research assistant then observed each participant in putting the monitor on properly and verified that the participants were able to record BP readings and confirmed successful data transmission to the mobile app. The enrollment session (including training) typically lasted about 30 min.

Participants then took the device home for one week, during which they were instructed to take 5 BP readings per 24 h cycle (once upon waking, once in the morning, once in the afternoon, once in the evening, and once in the middle of the night). Reminders for BP readings were programmed into each participant’s smartphone, either through the device’s associated mobile app or directly in the alarm function of the smartphone. The BP monitor also gathered information on physical activity (i.e., number of steps), sleep patterns, and heart rate throughout the time they were wearing the watch. Participants were asked not to remove the watch during this time period unless needed, such as for charging or taking a shower.

At the follow-up study visit (1 week after enrollment visit), the participant returned the smartwatch BP monitor. All data recorded by the monitor (BP, pulse, activity, and sleep) were exported into a secure electronic data capture database (REDCap). During this visit, they were asked to complete the System Usability Scale (SUS) Questionnaire [20,21], which measured the overall usability of the device, and the Post-Study System Usability Questionnaire (PSSUQ), which measured the user’s perceived satisfaction with the device and related mobile app. Both are standardized instruments for usability assessment in information technology [20]. We chose the SUS for its ability to measure overall usability and its frequent use in other studies of health information technology systems to enable comparisons in usability. However, it is not highly amenable to pinpointing specific issues in the product facing the user given the lack of individual domain scores. Therefore, we also included the PSSUQ since it includes different domains of usability (overall, system usefulness, information quality, and interface quality) [20] and because we did not want to rely solely on a single instrument for usability evaluation. A limitation of the PSSUQ is that it is limited to a 1-to-5 discrete scale and does not allow open-ended responses within the instrument itself, but we addressed this by soliciting open-ended responses separately. The full eHEALs questionnaire, SUS, and PSSUQ instruments are included in Appendix A, Appendix B and Appendix C, respectively. Participants also completed a follow-up questionnaire describing their experience with the monitor, whether it disrupted their other activities, and whether they would use the monitor outside the context of the research study (Appendix D). Data quality metrics were collected as another reflection of usability, such as the number of participants who had to discontinue the study, the number of participants who successfully completed all data collection measurements are requested, and the patterns/consistency of data collection.

In addition to the structured questionnaires and usability instruments, participants also provided narrative/unstructured open-ended responses regarding their experience using the devices.

### 2.3. Statistical Analyses

Descriptive statistics of the study cohort and of usability scores were generated using the mean and standard deviations or median and interquartile ranges depending on the distributions of continuous variables, or counts/frequencies for categorical variables. Variations in digital health literacy (eHEALS) and usability scores (SUS and PSSUQ) across various demographic groups were evaluated with analysis of variance (ANOVA) after distributions were verified to be normal. For results with significant variation across all categorical groups based on ANOVA, we planned to conduct post hoc tests consisting of multiple pairwise comparisons between groups (Tukey multiple comparisons of means) to discern which groups had mean differences that were statistically significant. For age, we treated this as a continuous variable instead of a categorical variable to enhance statistical power, but graphically depicted the data in categorical form to facilitate ease of interpretation. *p*-values less than 0.05 were considered statistically significant. Statistical analyses were conducted using Microsoft Excel (Microsoft Corporation, Redmond, WA, USA) for descriptive analyses and R software for ANOVA.

Open-ended responses were coded by two independent coders (SBB and MSB) for thematic content. Comments were iteratively reviewed and mapped to various thematic domains. Discrepancies in emerging themes were reviewed by all co-authors until a consensus was reached. Representative comments demonstrating the major themes, chosen and agreed upon by all co-authors, were extracted for illustration.

## 3. Results

### 3.1. Study Cohort Characteristics

Table 1 describes the demographic characteristics of the study cohort. At the time of analysis, we enrolled a total of 53 participants, 2 of whom (3.8%) discontinued the study and did not complete data collection. The remaining 51 participants contributed data and were included in subsequent analyses. The mean (standard deviation, SD) age was 66.1 (18.5) years. The majority (34/51, 67%) were female. The cohort was racially diverse: 29 (57%) were White, 11 (22%) were Black/African American, 9 (18%) were Asian, 1 (2%) was Native American/Alaskan Native, and 1 (2%) was more than one race.

### 3.2. Digital Health Literacy

**Figure 1.**
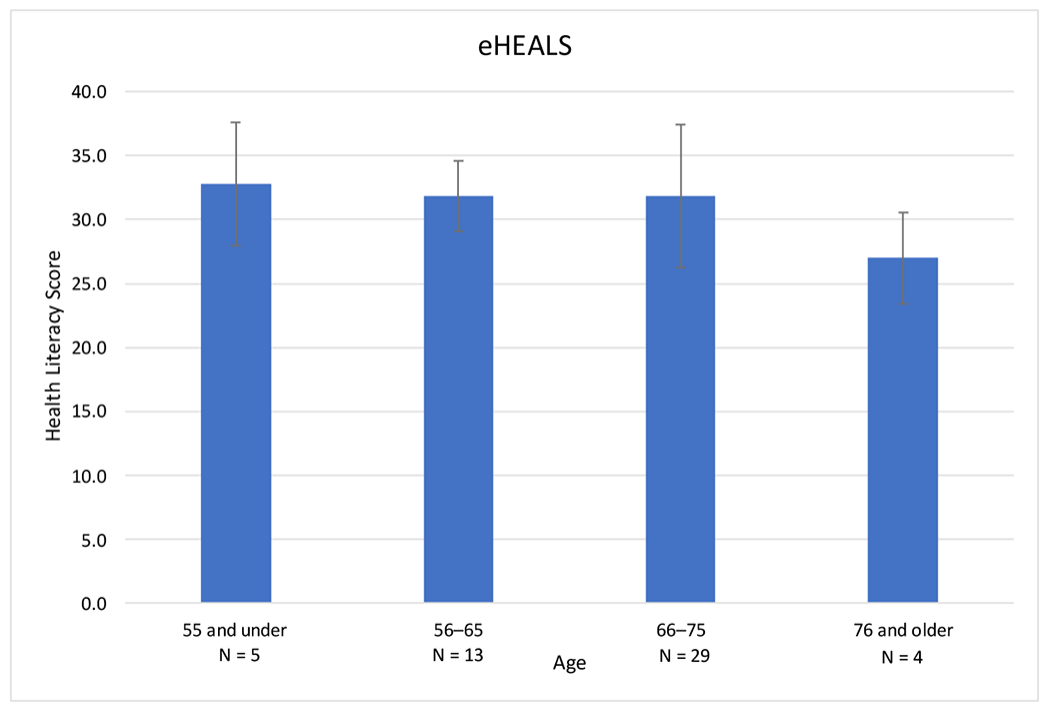
eHEALS digital health literacy score based on age in a cohort of glaucoma patients. Higher scores indicate greater self-perceived digital health literacy. Scores in this cohort revealed a downward trend in digital health literacy with increasing age, but did not reach statistical significance. Age was treated as a continuous variable in the model but depicted here categorically for ease of interpretability.

eHEALS measures electronic health literacy using eight standardized questions [19]. Scores fall on a scale of 8–40, with 8 being the lowest score and 40 being the highest. Higher scores correlate with higher perceived electronic health literacy. In this cohort, the mean (SD) eHEALS score was 31.6 (4.9) (Table 2). The range for eHEALS was 19 with a minimum of 21 and a maximum of 40. There was a downward trend in health literacy scores by age (Figure 1). With increasing age, the mean digital health literacy scores decreased from 32.8 (age 55 and under) to 27.0 (76 and older), but these differences were not statistically significant. There were no significant differences in digital health literacy scores based on gender or race in this cohort.

### 3.3. Usability Scores

We used two standardized instruments for assessing usability among adult patients with glaucoma using the home BP smartwatch monitor. The SUS is a standardized instrument with 10 items that asks a user to rate the usability of varying devices, such as an application or device [20,21]. The range of scores is 0–100, with higher scores indicating greater usability. The median (IQR) SUS score among study participants was 80 (17.5).

The PSSUQ is a standardized instrument with 16 standardized questions that are used to measure users’ perceived satisfaction at the end of a study [22]. The range of scores for this instrument is 1–7, with 1 relating to higher perceived satisfaction (i.e., lower scores reflect greater usability). The medians (IQR) for all four sections (overall PSSUQ, system usefulness, information quality, interface quality) of the PSSUQ among our study participants were 1.69 (1.59), 1.33 (1.42), 1.67 (2.08), and 1.67 (1.67), respectively. They all had a minimum of 1 and maximum of 7, and the IQR ranged from 1.6 to 2 for all sections.

**Figure 2.**
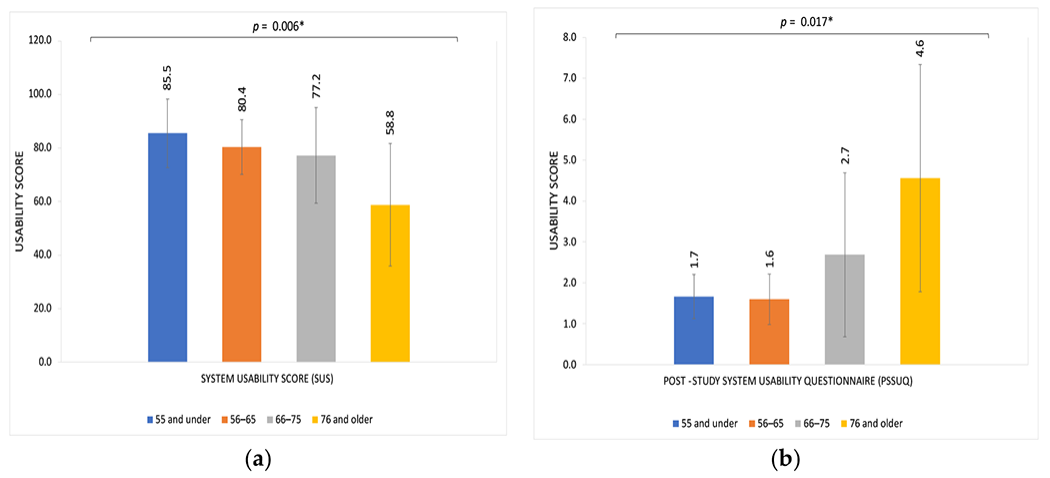
Usability scores of a smartwatch device for home BP monitoring by age group among a cohort of adults with glaucoma. (**a**) System Usability Score (SUS) in participants by age group; (**b**) Post Study System Usability Questionnaire (PSSUQ) in participants by age group. Higher SUS and lower PSSUQ scores reflect greater usability. Age was treated as a continuous variable in the model but depicted here categorically for ease of interpretability.

We examined variations in usability assessments based on various characteristics, including age, gender, and race (Table 3). Age was the only factor with a statistically significant association with variation in usability assessments of the home smartwatch BP monitor. Scores on both the SUS and PSSUQ reflected significantly decreased usability with older age, with mean SUS of 58.8 and mean PSSUQ of 4.6 among patients 76 years and older (*p* = 0.006 and *p* = 0.017 on ANOVA with age as a continuous variable, respectively; see Figure 2). There were no significant differences in usability scores based on gender or race, for either the PSSUQ or the SUS (Table 3).

The follow-up questionnaire that patients completed at their follow-up visit entailed a series of statements to which patients responded on a Likert scale, with 1 representing “not agree at all” to 10 representing “completely agree.” The questionnaire results showed a high level of agreement that the wearable smartwatch device was useful, with a mean (SD) rating of 7.8 (2.5) on a scale of 1–10. Patients generally disagreed that the monitor caused them discomfort (mean [SD] rating of 3.8 [3.1]) or disturbed their sleep 1.9 (2.9). Similarly, patients disagreed that the device negatively affected their everyday physical activities, with the mean (SD) rating being 1.5 (2.4). Even though many patients agreed that the device provided valuable information about their health and fitness 7.6 (2.8), they were neutral about using this device outside of the scope of the study 4.5 (4.1).

### 3.4. Data Quality Metrics

We also assessed usability of the system based on measures of data quality. During enrollment, participants were asked to take five BP measurements per day for 7 days. The vast majority (46/51, 90.2%) contributed some BP measurements on all 7 days; the few who did not still contributed BP measurements on 2–3 days. Approximately half (27/51, 52.9%) were able to record 4–5 BP measurements per day on all 7 days. The most frequently missing BP measurements were during the nighttime, with 9/51 (17.6%) not having any middle-of-the-night measurements.

### 3.5. Themes from Open-Ended Responses

Participants were asked to leave open-ended responses after their experience, and these were analyzed thematically (Table 4). Through analysis, it was found that the most common themes explained that patients found the device difficult to use at times, such as with the Bluetooth connectivity and how to ensure data were being transferred from the watch to the app. Themes also included that of the watch being too bulky and uncomfortable even though it has an adjustable strap, as well as having difficulty troubleshooting the device when measurements were not being taken. Although the device gives an error message, patients would oftentimes be unsure of what to fix in order to obtain a measurement. Regarding the lack of nighttime measurements, participants related difficulty remembering to set alarms, difficulty waking up in the middle of the night to measure BP, and not being willing to disturb their sleep to take BP measurements.

## 4. Discussion

In this study, we evaluated the usability of the first commercially available smartwatch capable of measuring BP among a cohort of adults with glaucoma by analyzing both structured questionnaires and open-ended feedback regarding their experiences using these devices at home. Our primary findings were that the device achieved high usability ratings among the overall cohort, but older age was associated with lower levels of reported usability/user experience. This highlights the promise of this technology for monitoring and risk stratification for patients with glaucoma, while also demonstrating an important gap meriting future improvements in design to enhance the effectiveness of informatics and digital health interventions for elderly populations.

This study was motivated by increasing interest in understanding the relationships between BP and glaucoma, particularly because BP management guidelines have evolved in recent years. This is largely due to clinical trials such as the Systolic Blood Pressure Intervention Trial (SPRINT) [23], which showed that intensive systolic BP control to less than 120 mmHg was significantly beneficial to overall health. Some investigators have posited that intensive BP control may be specifically beneficial to glaucoma patients because a decrease in BP of 10 mmHg within five years led to a decrease in IOP [24], a known risk factor for glaucoma. However, others have expressed concern that intensive BP management may actually increase the risk of hypotensive events (particularly nocturnal hypotensive events that may not be symptomatic or noticeable to patients) that can increase the risk of optic nerve hypoperfusion and subsequently glaucoma progression [25]. More studies are needed to understand how changes in BP may affect risk of glaucoma development and progression, and to do this, devices are needed to assess patients’ BP and record these data. Given that most patients with glaucoma are ambulatory outpatients, there is a need to understand devices designed for home use. Additionally, blood pressures taken in a clinical setting can be non-representative (with “white coat hypertension” being a well-known entity) and can additionally be particularly inaccurate for patients with hypertension [26]. With this need for home monitoring, and increasing adoption of smartwatch devices for health monitoring [27], we decided to evaluate the usability of the Omron HeartGuide monitor, the first commercially available smartwatch in the United States with BP monitoring capabilities.

First, we found that the device achieved high ratings of usability overall among the study cohort. Mean scores of 77.40 and 2.46 for the SUS and PSSUQ, respectively, indicate usability in the 80th–84th percentile [28]. Usability scores were even higher if focusing on the subgroups of younger/middle-aged patients, including those aged 56–65 years (Figure 2). To provide context, this far exceeds average usability scores for electronic health record (EHR) systems, which have been reported to have an average SUS score of 45.9 (21.9) amongst physicians, who have been trained in using the EHR [29]. Prior validation studies found the monitor to be quite accurate in BP measurements, typically within 2 mmHg of diastolic and systolic pressures measured with gold standard arm-cuff-based monitors. The accuracy of the device, coupled with highly rated usability among participants in their 50s and 60s, is encouraging. Because glaucoma development and progression can often occur insidiously over the course of many years, facilitating early detection and treatment is critical. Therefore, risk stratification and monitoring in these earlier age groups is high-yield, and the usability of the BP monitor in these groups is promising for future utility. Granular BP data from devices such as these may help to improve predictive models of glaucoma that have shown BP data to be important variables for risk stratification, and may help identify high-risk patients who need closer monitoring at earlier stages to mitigate the probability of disease progression [30,31].

However, increasing age was associated with decreased usability on both SUS and PSSUQ scores, indicating that older patients, particularly those over age 75, had significantly greater difficulty using the BP monitor watch studied. The digital divide between younger and older populations has been recognized for some time [32,33], and the COVID-19 pandemic highlighted some of these disparities [34]. These disparities are more pronounced now with technological advances in healthcare delivery, including increasing adoption of telehealth and digital health integration [35,36]. This is a reminder that though health data from wearable electronic devices have the potential to revolutionize precision medicine and patient involvement in their own care, efforts must be made to ensure these technologies are practical for older populations [34], as they carry a disproportionate burden of chronic disease (as is the case for glaucoma) and are likely to have the most potential benefit from their implementation. However, older adults face particular challenges with technology use, including a lack of familiarity (with only 27% of American adults ages 65 and older reporting smartphone ownership as recently as 2017 [37]), physical challenges (such as difficulty reading small print and joint/manual dexterity issues with handling small devices), and potential cognitive barriers. These challenges are further magnified in particular populations, such as those with vision-threatening eye conditions such as glaucoma, who may have particular challenges with technology accessibility. For these populations, strategies such as larger font size and magnification, text-to-voice, or voice-enabled functionality may prove especially useful. This emphasizes the need for device development using participatory design, which is a methodology that incorporates user input during the innovation process [35]. As a recognition of the particular design challenges of this population, an entire field of gerontechnology has emerged to specifically study the needs of older adults in technology design and implementation [38,39].

Beyond age-related challenges, usability issues reported for the wearable smartwatch BP monitor relayed similar themes to that of other BP measurement devices [40]. With the use of a traditional BP monitor, patients often complain about the fit of the cuff, similar to this study, where patients complained about the fit of the watch itself. Similarly, thematic analysis showed that participants in this study struggled to troubleshoot when the device reported errors on the screen, which is comparable to struggles patients face when using a traditional BP monitoring system [41].

This study is not without limitations. First, participants were derived from a single institution, thus potentially limiting generalizability, although a key strength of our study was the overall diversity of participants across age groups, gender, and racial/ethnic categories. Second, participants were given one week to use the device due to a limited number of available devices. Usability ratings among older adults may have possibly been improved over a longer follow-up interval, giving participants more time to learn and use this new device. However, the 7-day period of BP data collection represents a much higher frequency of data collection than typical outpatient clinical practice (which often consists of 1 daytime measurement every few months) or even of traditional ambulatory BP monitoring, which is performed over the course of a 24–48 h period [42]. We also did not specifically ask participants about the role of caregivers or family members in assisting them with use of the device. Finally, although there was training provided at enrollment and active verification of proper fit, BP measurement, and data transmission, it is possible that more intensive training may have improved usability among the older participants. However, our goal was to understand the experience of using the device in an “off-the-shelf”, real-world environment as much as possible.

In summary, we conducted detailed usability evaluations of a smartwatch device designed for home BP monitoring among a cohort of patients with glaucoma, finding overall high levels of usability demonstrating promise for future research studies and clinical implementation, but also finding some challenges among older adults. Glaucoma patients present unique considerations for digital health design, such as older age and visual impairment. This study provides an effective use case of evaluating usability concerns in a geriatric population managing a chronic disease that can impact their ability to engage and use technology. Understanding their digital health literacy and user experience is important for inclusive design and future interventions to improve health and vision outcomes.

## Figures and Tables

**Table 1.** Demographic characteristics of adult participants with glaucoma who piloted a smartwatch blood pressure monitor for home use (N = 51).

	Number (%)
Mean age (SD) in years	66.1 (8.5)
**Gender**	
Male	17 (33%)
Female	34 (67%)
**Ethnicity**	
Not Hispanic or Latino	46 (90%)
Hispanic or Latino	3 (6%)
Not Reported	2 (4%)
**Race**	
White	29 (57%)
Black/African American	11 (22%)
Asian	9 (18%)
Native American/Alaskan Native	1 (2%)
More than one race	1 (2%)

**Table 2.** eHealth Literacy Scale (eHEALS) scores for adult participants with glaucoma using a smartwatch blood pressure monitor at home by demographic group. eHEALS is an 8-item measure of eHealth literacy that measures consumers’ knowledge, comfort, and perceived skills in regard to electronic health information. Higher numeric scores indicate greater digital health literacy.

	Mean (SD) eHEALS Score	*p*-Value^a^
**Age**		0.11
55 and under	32.8 (4.8)	
56–65	31.8 (2.7)	
66–75	31.9 (5.6)	
76 and older	27.0 (3.6)	
**Gender**		0.83
Male	31.4 (5.6)	
Female	31.7 (4.6)	
**Race**		0.89
White	31.9 (4.5)	
Black/African American	31.3 (6.5)	
Asian	31.3 (4.8)	
American Indian/Alaskan	27.0 (0)	
More than one race	33.0 (0)	

a *p*-values were generated using ANOVA.

**Table 3.** SUS (System Usability Score) and PSSUQ (Post Study System Usability Questionnaire) scores for adult participants with glaucoma using a smartwatch blood pressure monitor at home by demographic group.

	Mean (SD) SUS Score		Mean (SD) PSSUQ Score	

		*p*-Value ^a^		*p*-Value ^a^
Age ^b^		0.006 **		0.017 **
55 and under	85.5 (12.8)		1.7 (0.5)	
56–65	80.4 (10.2)		1.6 (0.6)	
66–75	77.2 (17.8)		2.7 (2.0)	
76 and older	58.8 (22.9)		4.6 (2.8)	
Gender		0.94		0.90
Male	77.6 (21.1)		2.5 (2.0)	
Female	77.3 (14.7)		2.4 (1.8)	
Race		0.34		0.83
White	77.3 (16.0)		2.3 (1.8)	
Black/African American	76.1 (21.9)		2.4 (1.9)	
Asian	80.6 (10.4)		3.0 (2.3)	
American Indian/Alaskan	95.0 (0)		1.2 (0)	
More than one race	47.5 (0)		3.2 (0)	

a *p*-values were generated using ANOVA.

b Age was treated as a continuous variable in the model but depicted here categorically for ease of interpretability.

** *p* < 0.05

**Table 4.** Common themes and examples of comments and feedback from patients with glaucoma regarding their experiences of a smartwatch monitor designed for home blood pressure monitoring.

Themes	Comments
Lack of Technological Skill	“I had problems with pairing the device. I had to stop and reboot it frequently. Sometimes I’d have to try a few times to get a reading and needed to adjust my arm. I think part of it was that I am not that technically savvy.” “It was difficult to keep it charged.” “Not comfortable with installing apps or having to add information to the log sheet, and had a lot of error codes on the watch.” “Had difficulty using the app and monitor.”
Uncomfortable/Improper Fit	“Would be more likely to use if the device was smaller.” “Concerned about the durability of the interior of the cuff. It is flimsy and needs better durability.” Not used to wearing a watch and found it binding. “The watch is way too big.” “The monitor is size M and I think a small one would be better. My wrist is 5.8 inches. The monitor tended to slip around quite a lot. I have several ‘errors’ and ‘irregular heartbeats’ and am not sure if it is because it’s too big. The measurements were always quite different compared to my Omron cuff device.” “Watch is too chunky.” “The fit was not conducive, maybe a velcro band to make it more flexible.”
Unable to Troubleshoot	“It didn’t register all the steps.” “It did not register readings correctly, came up with code 2, talking, without talking.” “Too difficult to find the correct position for taking a measurement.” “The manual does not have a description and solution for all error codes. No clear instructions on how to take action in certain situations. The display also disappears too quickly.”

## Data Availability

The data presented in this study are available on request from the corresponding author. The data are not publicly available due to narrative free-text responses that may contain identifying information about participants.

## Appendix A. eHEALS Questionnaire

eHEALS (5 choices on a scale from strongly disagree to strongly agree)

1. I know what health resources are available on the internet.
2. I know where to find helpful health resources on the internet.
3. I know how to find helpful health resources on the internet.
4. I know how to use the Internet to answer my questions about health.
5. I know how to use the health information I find on the internet to help me.
6. I have the skills I need to evaluate the health resources I find on the internet.
7. I can tell high quality health resources from low quality health resources on the internet.
8. I feel confident in using information from the internet to make health decisions.

## Appendix B. SUS

SUS (5 choices on a scale from strongly disagree to strongly agree)

1. I think I would like to use this tool frequently.
2. I found the tool unnecessarily complex.
3. I thought the tool was easy to use.
4. I think that I would need the support of a technical person to be able to use this system.
5. I found the various functions in this tool were well integrated.
6. I thought there was too much inconsistency in this tool.
7. I would imagine that most people would learn to use this tool very quickly.
8. I found the tool very cumbersome to use.
9. I felt very confident using the tool.
10. I needed to learn a lot of things before I could get going with this tool.

## Appendix C. PSSUQ

PSSUQ (7 choices on a scale from strongly agree (=1) to strongly disagree (=7).

1. Overall, I am satisfied with how easy it is to use this system.
2. It was simple to use this system.
3. I was able to complete the tasks and scenarios quickly using this system.
4. I felt comfortable using this system.
5. It was easy to learn to use this system.
6. I believe I could become productive quickly using this system.
7. The system gave error messages that clearly told me how to fix problems.
8. Whenever I made a mistake using the system, I could recover easily and quickly.
9. The information (such as online help, on-screen messages, and other documentation) provided with the system was clear.
10. It was easy for me to find the information I needed.
11. The information was effective in helping me complete the tasks and scenarios.
12. The organization of information on the system screens was clear.
13. The interface of this system was pleasant.
14. I liked using the interface of this system.
15. This system has all the functions and capabilities I expect it to have.
16. Overall, I am satisfied with this system.

## Appendix D. Follow-Up Questionnaire

Follow-Up Questionnaire (1-10, not useful/minimal–very useful/maximal)

1. How useful was the information from the wearable smartwatch device?
2. How much discomfort did the device cause you?
3. How much did the device disrupt your sleep?
4. How much did this device disrupt your typical physical activities?
5. How valuable was the information provided by this device regarding your health or fitness?
6. How likely would you be to use the device outside the context of this research study?
